# Inhibitory effect of main phenolic acid components of *Jacobaea cannabifolia (Less.)* on inflammation caused by PM_2.5_

**DOI:** 10.3389/fphar.2022.1096137

**Published:** 2023-01-09

**Authors:** Bao-Li Xu, Yuan-Yuan Wang, Ling-Ling Jiang, Zhen Liu, Ding-Rui Liu, He Zhao, Shi-Liang Li, Xiao-Bo Wang

**Affiliations:** ^1^ 967th Hospital of People’s Liberation Army, Dalian, China; ^2^ Department of Pharmacy, Affiliated Zhongshan Hospital of Dalian University, Dalian, China

**Keywords:** PM_2.5_, *Jacobaea cannabifolia (Less.)*, PHBA, PHPAA, toll-like receptors

## Abstract

PM_2.5_ is an important environmental problem threatening human health at present, which poses serious harm to human body after inhalation. *J. cannabifolia* is a traditional Chinese medicine which exhibits anti-inflammatory effect. This study aimed to investigate the inhibitory effect of main phenolic acid components of *J. cannabifolia* on inflammation caused by PM_2.5_. Effect of PM_2.5_ on cell activity and apoptosis were determined by MTT, flow cytometry and calcein AM/PI staining. PHBA, PHPAA, and mixture of PHBA and PHPAA of different concentrations were given to RAW264.7 cells pretreated with PM_2.5_. The effect of drugs on cellular inflammatory factors was detected by ELISA. The expressions of TLRs related signal pathway at protein and gene levels were detected by western blot and qRT-PCR. The results showed that PM_2.5_ had no effect on cell activity and apoptosis within the determined concentration range. PHBA and PHPAA could markly inhibit the level of IL-1β, IL-6, and TNF-α in RAW264.7 cells. Furthermore, the expressions of TLR2, TLR4, MyD88, IRAK1, TRAF6, TAK1, IKKβ, and NF-κB induced by PM_2.5_ were markedly inhibited by PHBA and PHPAA at protein and gene levels. This study demonstrated that PHBA and PHPAA could attenuated inflammation caused by PM_2.5_ through suppressing TLRs related signal pathway.

## 1 Introduction

In recent years, some cities in China have experienced haze weather with particulate matter as the main pollution feature, and the ambient air quality is facing a severe test, which is extremely adverse to the national health and socio-economic development (Lin et al., 2018). PM_10_ (diameter of inhalable particles which are less than 10 μm) and PM_2.5_ (diameter of inhalable particles which are less than 2.5 μm and can enter the lung) are the main pollutant (Huang et al., 2014). The particle size of PM_2.5_ is smaller, resulting in a larger surface area, which makes it easy to be absorbed. At the same time, PM_2.5_ can stay in the atmosphere for a long time, and can travel a long distance (Han et al., 2015; Ji and Zhao., 2015). Therefore, PM_2.5_ has a great impact on people’s health and air quality.

The sources of PM_2.5_ are very complex, mainly including emissions of combustibles, particles generated in chemical processes, human activities, equipment operation, cleaning and cooking (Ji and Zhao., 2015; Li et al., 2016; Zhou et al., 2016). PM_2.5_ is a major threat to human health and can affect many systems of the body, including the central nervous system, blood system, metabolic immune system, urogenital system, digestive system and skin (Boothe et al., 2014; Park and Wang., 2014; Piao et al., 2018; Geng et al., 2019; Jeong et al., 2019; Shaffer et al., 2019). Therefore, it is very important for human health to develop new drugs to treat PM_2.5_.

**FIGURE 1:**
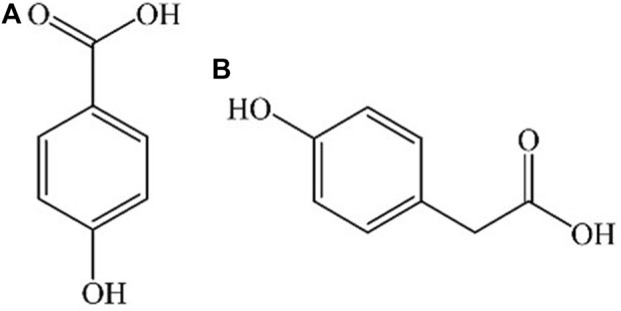
Chemical structures of **(A)** PHBA and **(B)** PHPAA.

*Jacobaea cannabifolia (Less.) E.Wiebe* is a plant of *Senecio* genus, which is a genuine medicinal material in Northeast China. *J. cannabifolia* has anti-inflammatory, bacteriostatic, anti-virus, immune regulation, anti-tumor, and other effects (Li et al., 2005; Chen et al., 2015). It is clinically used to treat acute and chronic bronchitis, asthmatic bronchitis and acute respiratory tract infection. The main metabolites of the herb are flavonoids, phenolic acids, alkaloids, volatile oils, glycosides, and tannins. P-hydroxybenzoic acid (PHBA) and p-hydroxyphenylacetic acid (PHPAA) (Figure 1) are the main phenolic acids in *J. cannabifolia* and they have anti-inflammatory and other activities (Zhao et al., 2013; Sun et al., 2014).

This study aimed to explore the effect of PHBA and PHPAA on inflammation induced by PM_2.5_ and its possible mechanism, to provide an experimental basis for PHBA and PHPAA to become clinical therapeutic drugs for lung injury caused by PM_2.5_.

## 2 Materials and methods

### 2.1 Chemicals and reagents

PHBA (≥99%) and PHPPA (98%) were provided by Sigma Chemical Co. (St. Louis, MO, United States). PM_2.5_ particles were provided by Hong-Hai Yu, senior engineer of Huadian Electric Power Research Institute Co., Ltd. Northeast Branch. Mouse tumor necrosis factor-α (TNF-α), interleukin-6 (IL-6) and interleukin-1β (IL-1β) ELISA kits were purchased from Biyuntian Biotechnology Research Institute. TLR4, TLR2, TRAF6, NF-κB, MyD88, IRAK1, TAK1, and IKKβ polyclonal antibody IgG were obtained from Proteintech Group Inc (Chicago, IL, United States).

### 2.2 Cell culture

RAW264.7 murine macrophage cells were purchased from cell bank of Chinese Academy of Science, Shanghai. Cells were maintained in Dulbecco’s modified Eagle’s medium (Hyclone, Logan, UT) supplemented with 5% FBS (Gibco, United States), 1% streptomycin and penicillin (Hyclone) at 37°C in 5% CO_2_ in a humidified atmosphere incubator.

### 2.3 Determination of cell viability

The cell viability was determined by MTT method after treating with PM_2.5_. Collect RAW264.7 cells growing in logarithmic phase, add culture medium to make cell suspension, adjust cell concentration, and add 100 μl cell suspension to each well, and adjust the cell density to 1.0 × 10^4^/well. The cells were incubated in an environment of 5% CO_2_ and 37°C. When the cells covered the bottom of 96 wells, a series of concentrations of PM_2.5_ were added. After incubation for 48 h, add 10 μl MTT to each hole, culture the cells for 4 h, discard the culture medium, and carefully rinse it out with PBS for 2–3 times, then add 100 μl DMSO to each hole. Vibrate the shaking table at a low speed for 10 min, set the OD value to 490 nm on the microplate reader and conduct a light absorption measurement for each test hole, and calculate the cell survival rate. Survival % = (A490 nm for treated cells/A490 nm for control cells) × 100%, where A490 nm represents the absorbance value.

### 2.4 Annexin V-FITC/PI apoptosis assay

RAW264.7 cells were pretreated with PM_2.5_ (0, 10, 20, 40, 80, 100 μg/ml) for 48 h. Cells were digest with trypsin, collected by centrifugation and washed twice with PBS. 1 × 10^6^ cells were suspended in 500 μl binding buffer followed by the addition of 10 μl Annexin V-FITC. After incubation at room temperature in dark for 15 min, add 5 μl PI and the cells were then analyzed using a flow cytometer (BD Accuri, US).

### 2.5 Calcein AM/PI staining

RAW264.7 cells were seeded in a 6-well plate at a density of 1 × 10^6^cells/well and cultured overnight in a 5% CO_2_ incubator at 37°C to adhere. The cells were incubated with different concentrations of PM_2.5_ for 48 h. According to the instructions, the cells were gently washed with PBS twice. A small amount of about 200 μl suspension was added to the test tube, and then an appropriate amount of 100 μl dye solution was added. Incubate the cell suspension at 37°C for 15 min and then the fluorescence microscope was used to observe the cell staining.

### 2.6 Determination of cytokines by ELISA

Determination of the effect of PM_2.5_ on the production of inflammatory factors in RAW264.7 cells by ELISA. RAW264.7 cells were seeded in a 96-well plate at a density of 1 × 10^4^cells/well and cultured overnight in a 5% CO_2_ incubator at 37°C. After cell adhesion, different concentrations of PM_2.5_ (0, 10, 20, 40, 80, and 100 μg/ml) were added into the culture medium and the cells were continued to culture for 48 h. Then, the concentration of IL-1β, IL-6, and TNF-α in each was measured according to the ELISA instruction with a microplate reader at 450 nm. The effects of PHBA and PHPAA on inflammatory cytokines induced by PM_2.5_ were also determined using ELISA method and the duration of drugs was 24 h. The concentrations of PHBA or PHPAA were 10, 100, and 1,000 μM. In the mixture of PHBA and PHPAA, the molar ratio was 1:1.

### 2.7 Quantitative real-time PCR

RAW264.7 cells (2 × 10^6^ cells/well) were seeded in a 6-well plate, pretreated with PM_2.5_ (80 μg/L) for 48 h, and then treated with PHBA, PHPAA or mixture of PHBA and PHPAA for 24 h. Trizol reagent (Invitrogen Life Technologies, Carlsbad, CA, United States) was used to extract the total RNA of the cells pretreated with PM2.5. Detection of mRNA expression with Applied Biosystems ViiATM 7 Real-Time PCR system. cDNA synthesis was performed using a PrimeScript™ RT Reagent Kit according to the manufacturer’s instructions. After reverse transcription, the cDNA was amplified using SYBR-Green Premix (Takara, Otsu, Japan). Level of TLR2, TLR4, MyD88, IRAK1, TRAF6, TAK1, IKKβ, and NF-κB mRNA expressions were examined, and GAPDH was used as an internal control. Data were analyzed by the 2^−ΔΔCT^ method. Sequences of primers used for qRT-PCR are list in Table 1.

**TABLE 1:** Sequences of primers used for qRT-PCR.

Gene	Strand	The sequence of the primer (5′-3′)	bp
TLR2	Forword	GAG​CAT​CCG​AAT​TGC​ATC​ACC	174
	Reverse	CCC​AGA​AGC​ATC​ACA​TGA​CAG​AG	
TLR4	Forword	CAT​GGA​TCA​GAA​ACT​CAG​CAA​AGT​C	179
	Reverse	CAT​GCC​ATG​CCT​TGT​CTT​CA	
NF-κB	Forword	GAA​GCC​GCT​GAC​CAT​GGA​A	103
	Reverse	GAT​CAC​AGC​CAA​GTG​GAG​TGG​A	
MyD88	Forword	TAC​AGG​TGG​CCA​GAG​TGG​AA	119
	Reverse	GCA​GTA​GCA​GAT​AAA​GGC​ATC​GAA	
IRAK1	Forword	CGG​ACT​TCC​ACA​GTT​CGA​GGT​A	125
	Reverse	TGA​CCA​GCA​AGG​GTC​TCC​AG	
TRAF6	Forword	TCA​TTA​TGA​TCT​GGA​CTG​CCC​AAC	150
	Reverse	TTA​TGA​ACA​GCC​TGG​GCC​AAC	
TAK1	Forword	AGCAGAGTAGCTGCGGT	134
	Reverse	GAGGAGCTTGCTGCAGAT	
IKKβ	Forword	CAG​AAT​CAT​CCA​TCG​AGA​CCT​GAA	122
	Reverse	TGC​ACA​GAC​TGC​CCT​GAT​CC	
GAPDH	Forword	TGT​GTC​CGT​CGT​GGA​TCT​GA	150
	Reverse	TTG​CTG​TTG​AAG​TCG​CAG​GAG	

### 2.8 Western blot

After overnight culture in a 6-well plate (2 × 10^6^ cells/well, 3 ml medium/plate), the cells were pre-treated with PM2.5 for 48 h and drugs for an additional 24 h, cells were harvested and lysed in lysis buffer for 15 min on ice. After incubation, lysates were centrifuged and supernatant was collected. The protein concentration was measured by the bicinchoninic acid (BCA) method. 20 μg protein sample was separated on 10% SDS-PAGE and transferred to PVDF membranes. At room temperature, the membrane was sealed with 5% skimmed milk powder dissolved in triple buffered saline containing .1% Tween-20 (TBST) for 1 h. Then incubate the membrane with the primary antibody that recognized TLR2, TLR4, MyD88, IRAK1, TRAF6, TAK1, IKKβ, NF-κB, and GAPDH in a shaking incubator at 4°C overnight, wash it with TBST three times, and incubate it with the secondary antibody conjugated with peroxidase diluted in the closed solution at room temperature for 1 h. After washing, proteins of interest were detected using ECL detection reagent.

### 2.9 Statistical analysis

Statistical analyses were performed using the SPSS 24.0 software. The data were expressed as the Mean ± SD. Multiple comparisons were evaluated by one-way analysis of variance (ANOVA) with Dunnett’s posttest. Statistical significance was accepted at *p* < .05 or *p* < .01.

## 3 Results

### 3.1 Effect of PM_2.5_ on RAW264.7 cells

**FIGURE 2:**
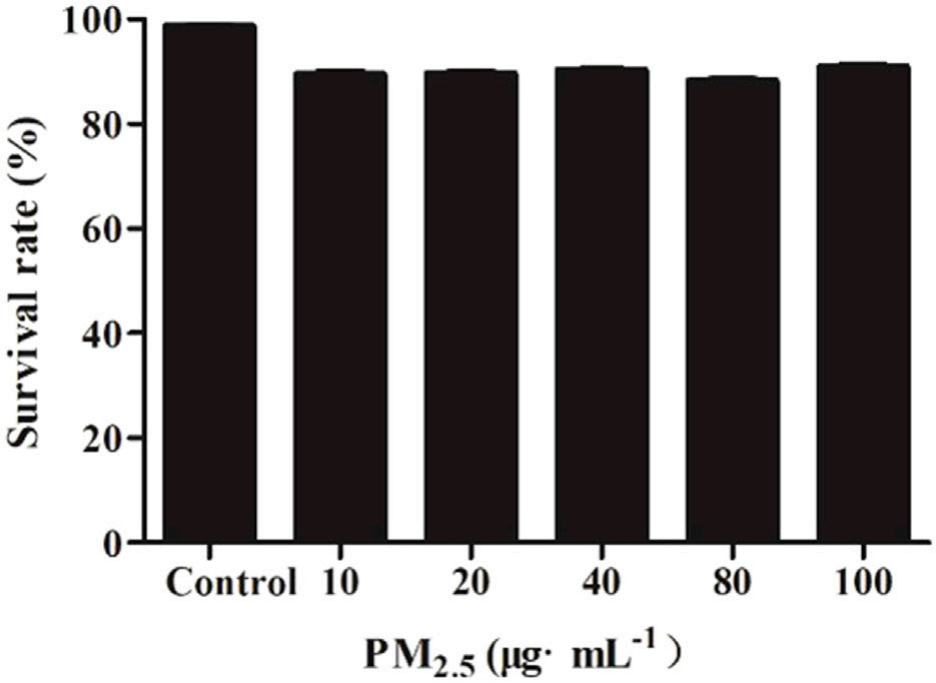
The cell survival rate caused by PM_2.5_.

**FIGURE 3:**
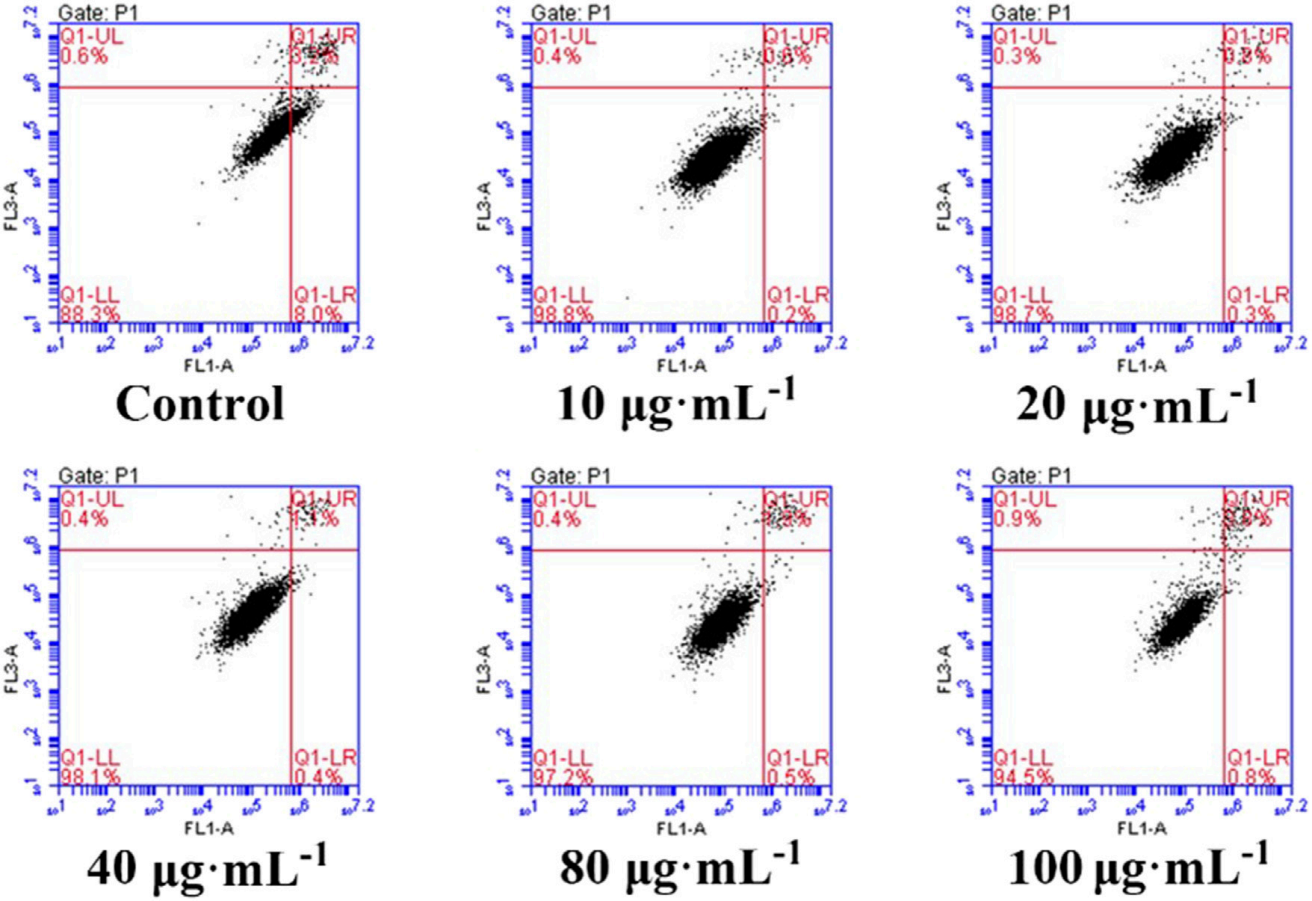
Effects of PM_2.5_ on apoptosis.

**FIGURE 4:**
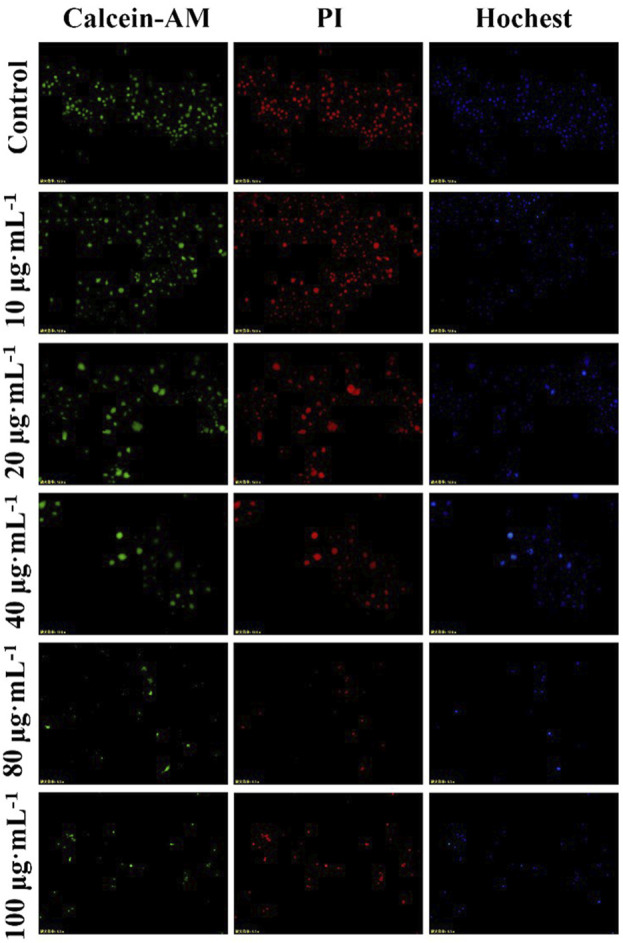
Calcein-AM/PI staining results.

After administration of PM_2.5_ at different concentrations, the survival rate of RAW264.7 cells decreased, but there was no statistical difference compared with the control group, indicating that PM_2.5_ within the measurement range could not inhibit the growth of RAW264.7 cells (Figure 2). It was found by flow cytometry that compared with the control group, PM_2.5_ of all concentrations did not induce apoptosis (Figure 3). Fluorescence staining results showed that the number of dead cells (red fluorescence) did not increase significantly after the action of PM_2.5_ of various concentrations. Hochest staining results showed that the nuclear morphology of each PM_2.5_ group did not change significantly, and no apoptotic cells were observed (Figure 4).

### 3.2 Effect of PM_2.5_ on inflammatory cytokines produced by RAW264.7 cells

**FIGURE 5:**
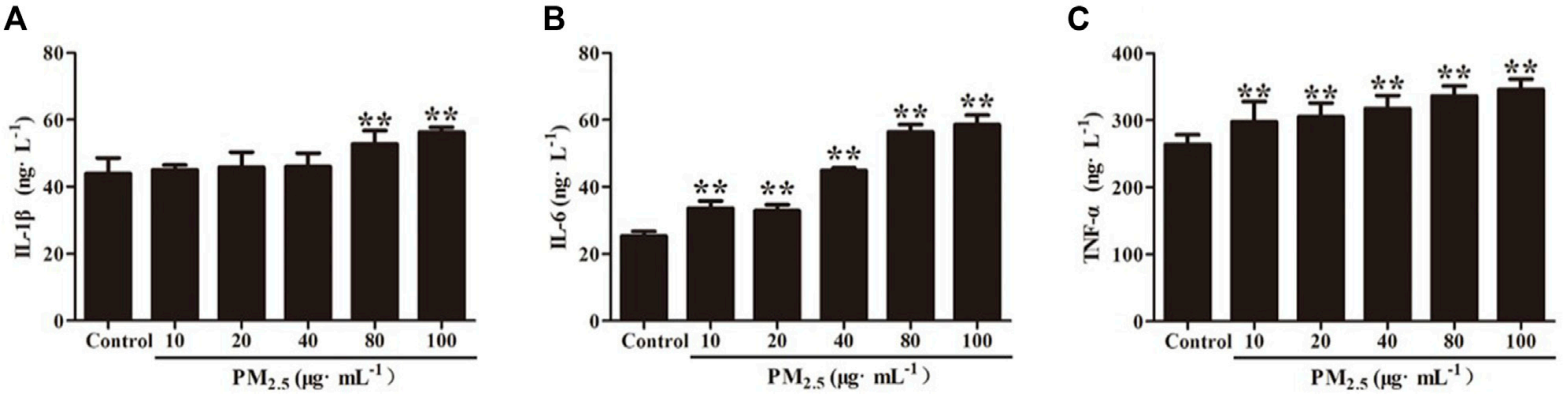
Inflammatory cytokine levels of **(A)** IL-1β, **(B)** IL-6 and **(C)** TNF-α in RAW264.7 cells stimulates by PM_2.5_. Values are means ± SD from three separate experiments. Compared with control group, **p < .01.

The inflammatory factors produced by RAW264.7 cells stimulated by PM_2.5_ were detected by ELISA, and the results showed that 80 μg/ml and 100 μg/mL PM_2.5_ produced significantly higher IL-1β, IL-6, and TNF- α than that of the control group (*p* < .01). Compared with the control group, there was no difference of IL-1β concentration produced by RAW264.7 cells that stimulated by 10, 20 or 40 μg/mL PM_2.5_(*p*>.05) (Figure 5). Through microscopic observation, high concentration of PM_2.5_ will cause unclear observation field of vision, so 80 μg/ml of PM_2.5_ was applied in subsequent experiments.

### 3.3 Inhibitory effect of PHBA and PHPAA on inflammatory factors produced by PM_2.5_

**FIGURE 6:**
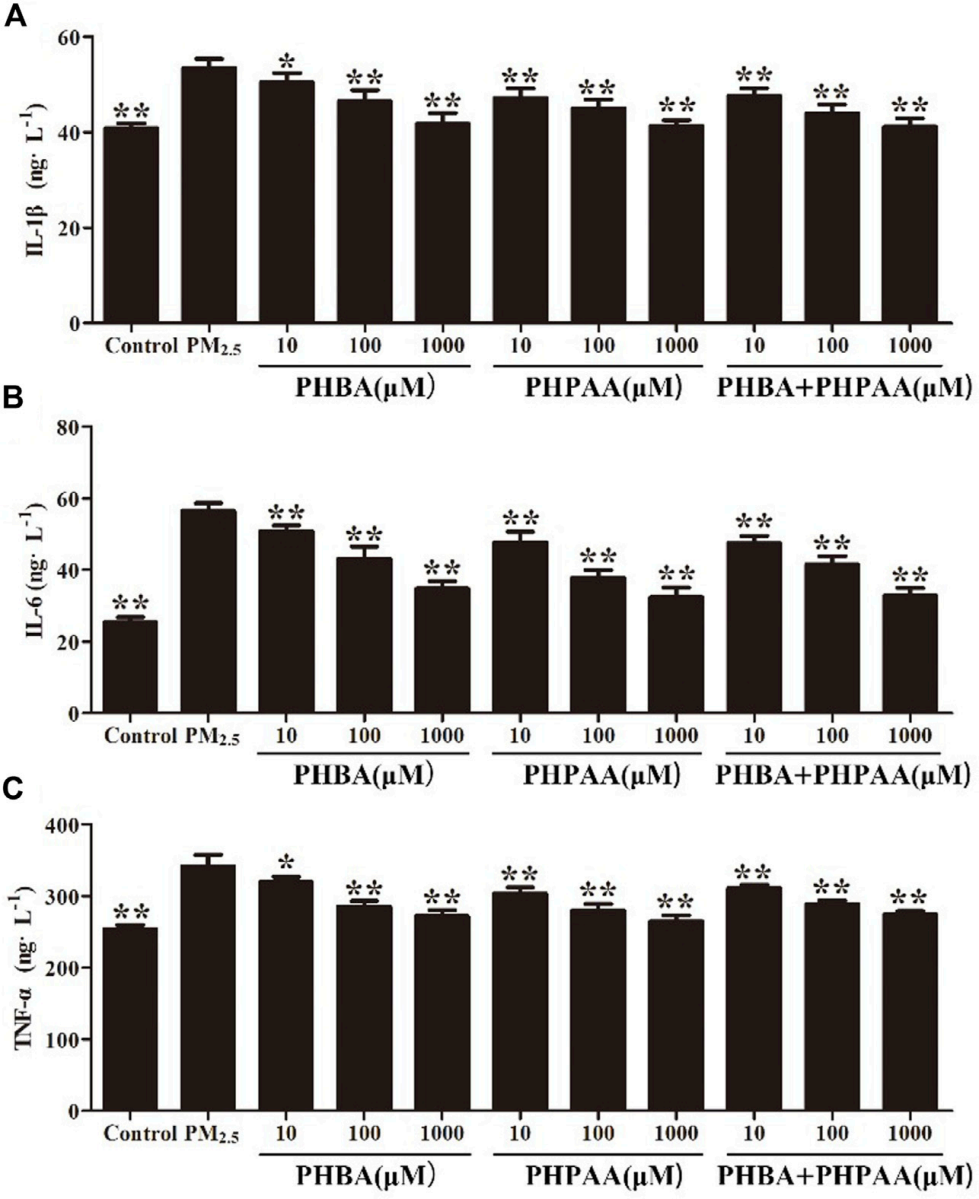
Inhibition of PHBA and PHPAA on inflammatory cytokines produced by PM_2.5_. **(A–C)**, Relative levels of IL-1β, IL-6, and TNF-α levels at 48 h assessed by ELISA. Values are means ± SD from three separate experiments. Compared with PM_2.5_ stimulation group, *p < .05, **p < .01.

Compared with the control group, PM_2.5_ significantly increased the production of inflammatory factors (*p* < .01). After different concentrations of PHBA or PHPAA (10, 100, 1,000 μM) treatment, the content of IL-1 β, IL-6, and TNF- α significantly decreased (*p* < .01). After combined treatment of PHBA and PHPAA (the molar ratio of PHBA and PHPAA was 1:1), the content of IL-1 β, IL-6, and TNF- α significantly decreased (*p* < .01) (Figure 6).

### 3.4 Effect of PHBA and PHPAA on TLRs related signal pathway protein expression

**FIGURE 7:**
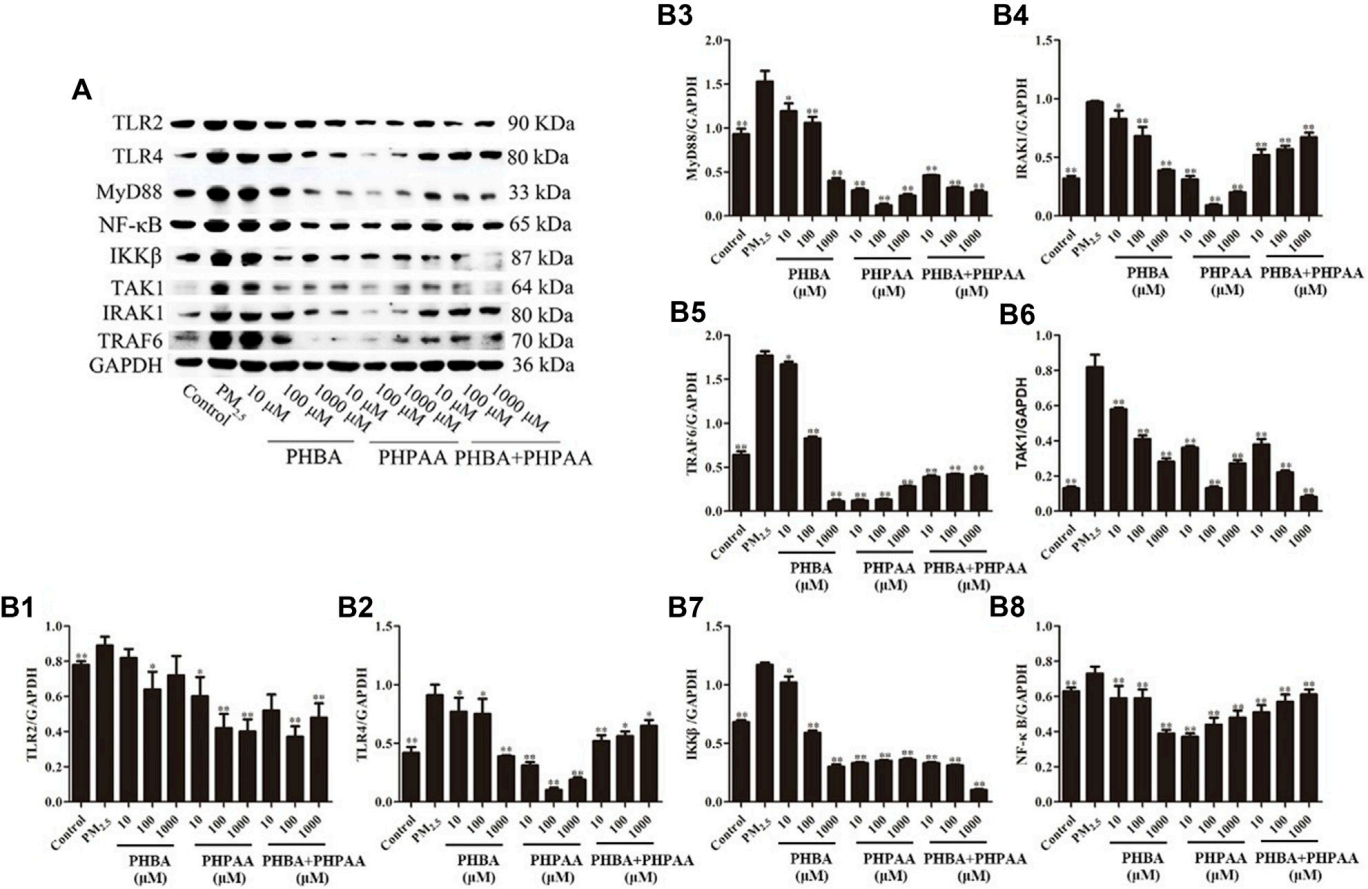
Protein expression of TLRs related pathways. **(A)**, Representative immunoblots of TLR2, TLR4, MyD88, IRAK1, TRAF6, TAK1, IKKβ, and NF-κB. **(B1–B8)**, WB results of TLR2, TLR4, MyD88, IRAK1, TRAF6, TAK1, IKKβ, and NF-κB in cell at 48 h. Values are means ± SD from three separate experiments. Compared with PM_2.5_ stimulation group, *p < .05, **p < .01.

Compared with the control group, the protein expression of TLRs (TLR2 and TLR4) and related receptors MyD88, IRAK1, TRAF6, TAK1, IKKβ, and NF-κB increased significantly after PM_2.5_ stimulation (*p* < .01) (Figure 7). After treating RAW264.7 cells stimulated by PM_2.5_ with different concentrations of PHBA, the expressions of TLR2, TLR4, MyD88, IRAK1, TRAF6, TAK1, IKKβ, and NF-κB decreased, and the changes showed a dose-effect relationship. However, when treated with a high concentration of PHBA (1,000 μM), the protein expression of TLR2 increased (Figure 7). Treating RAW264.7 cells stimulated by PM_2.5_ with different concentrations of PHPAA (10, 100, 1,000 μM) could make the protein expression of TLR2, TLR4, MyD88, IRAK1, TRAF6, TAK1, IKKβ, and NF-κB significantly decreased (*p* < .01). The protein expressions of TLR4, MyD88, IRAK1, TRAF6, TAK1, IKKβ, and NF-κB were increased when PHPAA was treated with high concentration (1,000 μM) (Figure 7). The combination of PHBA and PHPAA (1:1) also significantly decreased the expression of TLRs pathway proteins (*p* < .01), but increased the expression of TLR2, TLR4, IRAK1 and NF-κB proteins at high concentrations (1000 μM) (Figure 7).

### 3.5 Effects of PHBA and PHPAA on TLRs signal pathway gene expression

**FIGURE 8:**
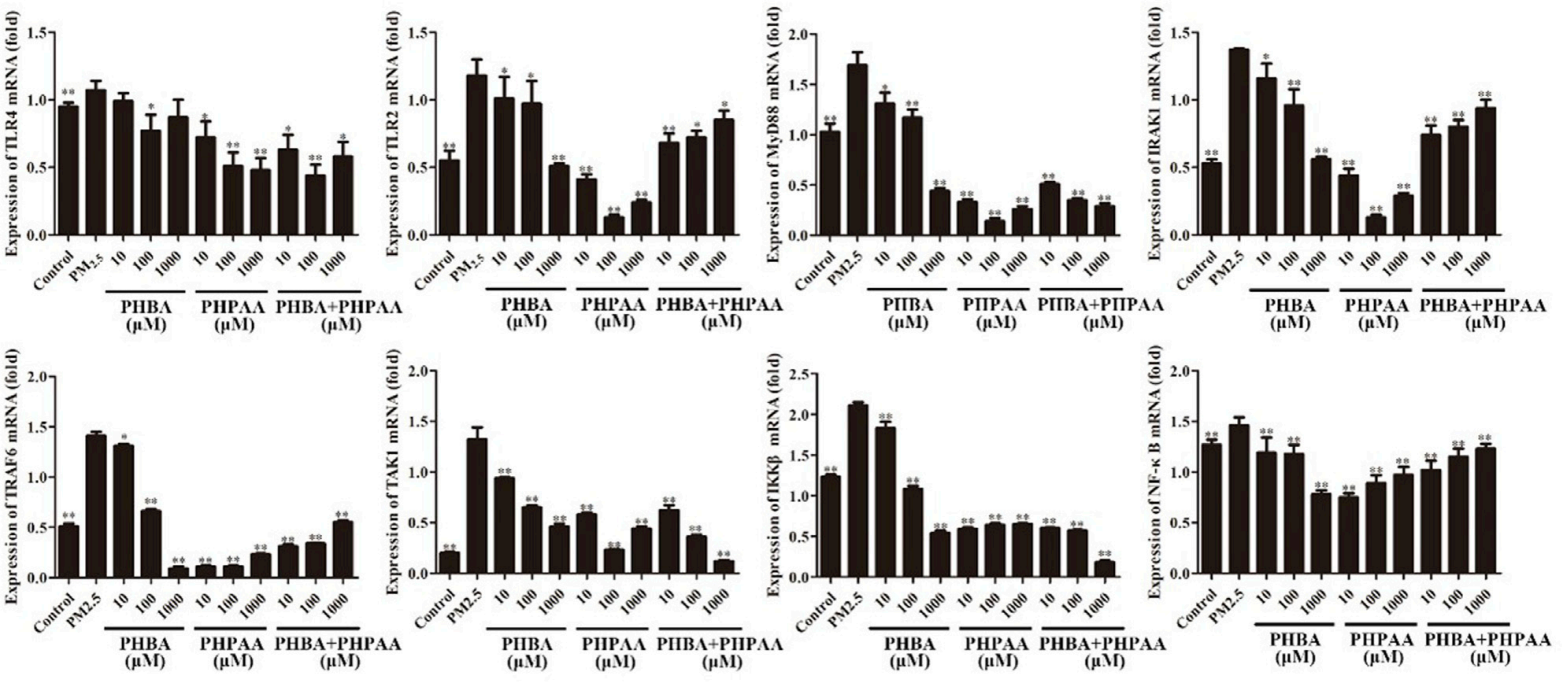
Gene expression of TLRs related pathways. Values are means ± SD from three separate experiments. Compared with PM_2.5_ stimulation group, *p < .05, **p < .01.

The gene expressions of TLRs and its downstream related pathway were determined by RT-qPCR. PM_2.5_ can significantly up-regulate the gene expression of TLR2, TLR4, MyD88, IRAK1, TRAF6, TAK1, IKK *ß* and NF- κ B(*p*<.01) (Figure 8, Table2). After treating RAW264.7 cells stimulated by PM_2.5_ with drugs for 24 h, the TLRs and its downstream pathway related genes were detected. The results showed that PHBA could significantly down regulate the TLRs related pathway genes in a dose effect relationship (*p* < .01) (Figure 8; Table2). After the cells stimulated by PM_2.5_ were treated with PHPAA, the gene expression of TLRs pathway was significantly decreased (*p* < .01). When PHPAA concentration is high (1,000 μM), the expression of related genes TLR4, MyD88, IRAK1, TRAF6, TAK1, IKK ß, and NF- κB increased (Figure 8; Table3). When the mixture of PHBA and PHPAA (1:1) was used, the gene expression of TLRs pathway was significantly decreased (*p* < .01). When the mixture is at high concentration (1,000 μM), the expression of TLR2, TLR4, IRAK1 and NF-κB significantly increased (Figure 8; Table4).

**TABLE 2:** Effects of PHBA on gene expression in TLRs related pathways.

Name	Control	PM_2.5_	PHBA(μM)		
			10	100	1,000
TLR2	.95 ± .03**	1.07 ± .07	.99 ± .06	.77 ± .12*	.87 ± .13
TLR4	.55 ± .073**	1.18 ± .12	1.01 ± .16*	.97 ± .17*	.51 ± .02**
MyD88	1.03 ± .08**	1.69 ± .13	1.31 ± .11*	1.17 ± .08**	.44 ± .03**
IRAK1	.53 ± .03**	1.37 ± .01	1.16 ± .11*	.96 ± .12**	.56 ± .02**
TRAF6	.51 ± .03**	1.41 ± .04	1.34 ± .02*	.66 ± .02**	.09 ± .02**
TAK1	.20 ± .01**	1.32 ± .12	.94 ± .01**	.65 ± .02**	.46 ± .03**
IKKβ	1.23 ± .03**	2.11 ± .04	1.83 ± .08*	1.08 ± .04**	.54 ± .03**
NF-κB	1.27 ± .05**	1.46 ± .08	1.19 ± .15**	1.18 ± .09**	.78 ± .04**

Note: Compared with PM_2.5_ stimulation group, *p < .05, **p < .01.

**TABLE 3:** Effects of PHPAA on gene expression in TLRs related pathways.

Name	Control	PM_2.5_	PHPAA (μM)		
			10	100	1,000
TLR2	.95 ± .03**	1.07 ± .07	.72 ± .12*	.51 ± .1**	.48 ± .09**
TLR4	.55 ± .073**	1.18 ± .12	.41 ± .04**	.13 ± .02**	.24 ± .02**
MyD88	1.03 ± .08**	1.69 ± .13	.33 ± .03**	.14 ± .03**	.26 ± .03**
IRAK1	.53 ± .03**	1.37 ± .01	.44 ± .05**	.13 ± .02**	.29 ± .02**
TRAF6	.51 ± .03**	1.41 ± .04	.11 ± .01**	.11 ± .01**	.23 ± .01**
TAK1	.20 ± .01**	1.32 ± .12	.58 ± .02**	.23 ± .01**	.44 ± .02**
IKKβ	1.23 ± .03**	2.11 ± .04	.59 ± .02**	.64 ± .02**	.65 ± .01**
NF-κB	1.27 ± .05**	1.46 ± .08	.75 ± .04**	.89 ± .08**	.97 ± .08**

Note: Compared with PM_2.5_ stimulation group, *p < .05, **p < .01.

**TABLE 4:** Effects of PHBA combined with PHPAA on gene expression in TLRs related pathways.

Name	Control	PM2.5	PHBA + PHPAA (μM)		
			10	100	1,000
TLR2	.95 ± .03**	1.07 ± .07	.63 ± .11*	.44 ± .08**	.58 ± .11*
TLR4	.55 ± .07**	1.18 ± .12	.68 ± .07**	.72 ± .05*	.85 ± .07*
MyD88	1.03 ± .08**	1.69 ± .13	.51 ± .02**	.35 ± .02**	.29 ± .03**
IRAK1	.53 ± .03**	1.37 ± .01	.74 ± .07**	.8 ± .05**	.94 ± .06**
TRAF6	.51 ± .03**	1.41 ± .04	.31 ± .02**	.34 ± .01**	.55 ± .02**
TAK1	.20 ± .01**	1.32 ± .12	.62 ± .05**	.36 ± .02**	.12 ± .01**
IKKβ	1.23 ± .03**	2.11 ± .04	.60 ± .01**	.57 ± .01**	.18 ± .02**
NF-κB	1.27 ± .05**	1.46 ± .08	1.02 ± .09**	1.15 ± .08**	1.23 ± .05**

Note: Compared with PM_2.5_ stimulation group, *p < .05, **p < .01.

## 4 Discussion

PM_2.5_ has a great impact on the central nervous system, blood system, metabolic immune system, digestive system, skin and other human bodies, and may lead to a variety of diseases. PM_2.5_ can destroy the integrity of the blood brain barrier (BBB) and make it easy for peripheral systemic inflammation to pass through the BBB to reach the central nervous system (Shou et al., 2019). The study found that the concentration of PM_2.5_ had a certain toxic effect on children’s bone marrow stromal cells, which affected the hematopoietic microenvironment of bone marrow. PM_2.5_ may activate PINK1/Parking pathway signal and induce mitochondrial autophagy by increasing ROS, and further activate HSCs (hepatic stellate cells) to cause liver fibrosis (Qiu et al., 2019). Cholesterol and squalene are key substances that affect the skin barrier function. PM_2.5_ can cause barrier disorder by increasing cholesterol synthesis, leading to the temporary accumulation of epidermal cholesterol and the reduction of squalene (Liao et al., 2019). It can be seen that PM_2.5_ is harmful to human health. It is very important to find effective drugs to treat PM_2.5_ as soon as possible.

It has been reported that PM_2.5_ can activate the inflammatory axis of vascular endothelial cell COX-2/PGES/PGE2, and promote cell apoptosis and inflammatory response (Yin et al., 2017). PM_2.5_ may induce apoptosis by increasing lipid accumulation, ROS level and activating mitochondrial pathway of macrophages. However, the results of MTT, flow cytometry and Calcein AM/PI staining showed that PM_2.5_ used in our study had no cytotoxicity to RAW264.7 and had no effect on apoptosis. The experimental results obtained in this study are different from those in related papers, which may be related to the physical and chemical properties of PM_2.5_ collected, processing and preservation methods of PM_2.5_.

It is reported that PM_2.5_ can cause airway inflammation and lung injury in mice, and can also produce cellular inflammation and secrete inflammatory factors from RAW264.7 cells (He et al., 2017; Ogino et al., 2018). In this study, PM_2.5_ was used to stimulate RAW264.7 cells, and the related inflammatory factor IL-1β, IL-6, and TNF-α secreted by cells significantly increased. However, when PHBA, PHPAA, the mixture of PHBA and PHPAA (1:1) were given in advance, the detection results of inflammatory factors secreted by cells were significantly reduced. It suggests that PHBA and PHPAA can repair the damage caused by PM_2.5_ through inhibiting inflammatory factors.

**FIGURE 9:**
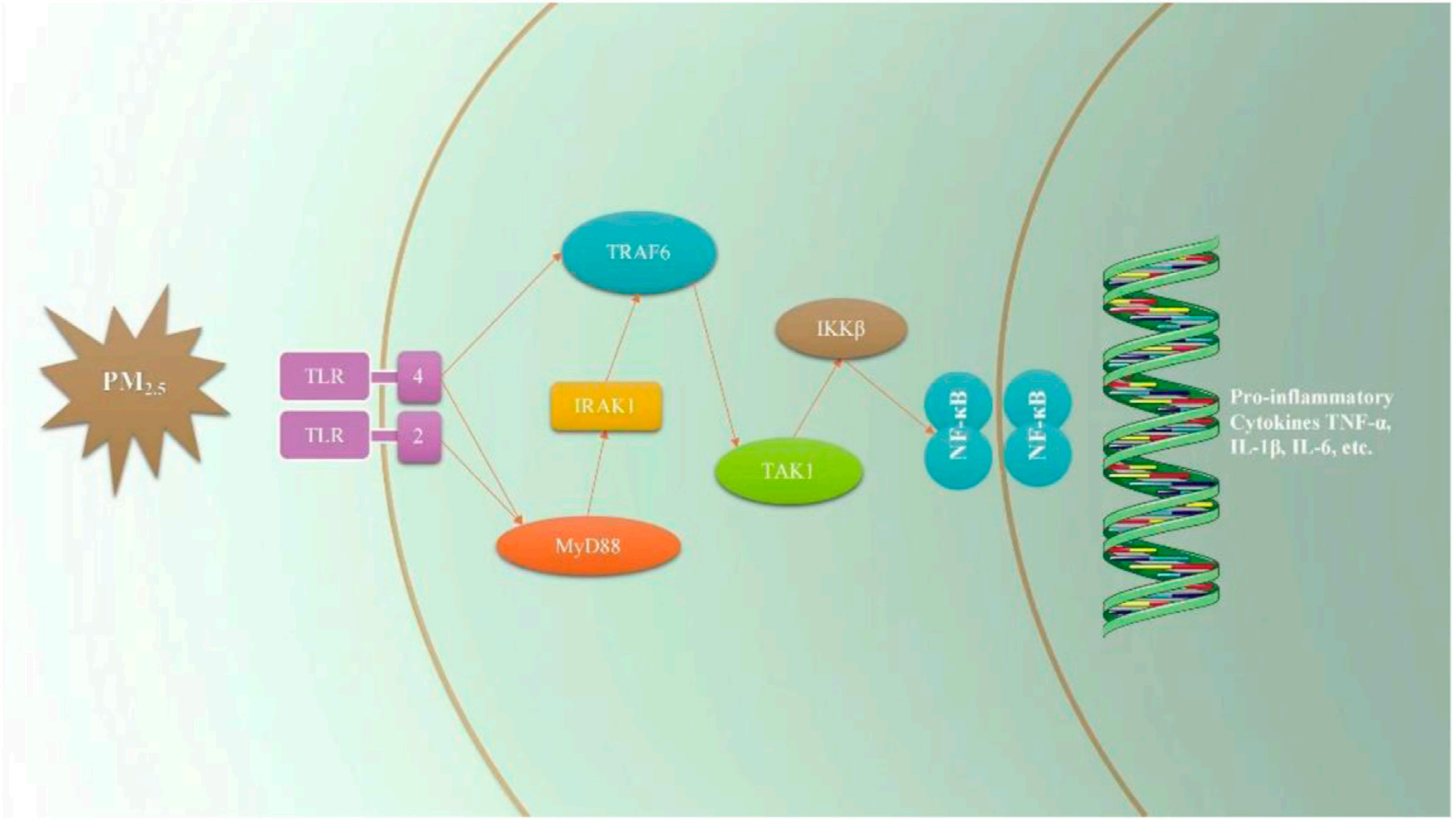
Sketch of the effect of PM_2.5_ on TLRs signal pathway.

In the induction of inflammatory response, TLRs play a role mainly through MyD88 and TRIF mediated pathways (Deguine and Barton., 2014). When sensing external stimuli, TLRs allow MyD88 to dock with MAL (MyD88 adapter-like). MAL is a bridging linker, often involved in TLR4 signal pathway, while TLR2 signal pathway is less involved, and interacts with MyD88 through TIR. In addition to the TIR domain, MyD88 also contains a dead domain, which can help it interact with IRAK4 (Janssens and Beyaert., 2003; Lim and Staudt., 2013). The interaction between these domains produces a large polymer, and the nitration of this polymer leads to the activation and dimerization of TRAF6 (Lin et al., 2010). TRAF6 mediates the ubiquitination of TAK1. The lack of TAK1 reduces the inflammatory signal of TLRs, but this phenomenon is not observed when TAB protein is lacking. TAK1 signal then activate NF- κ B and MAPK, respectively. NF-κB is a molecular center of inflammation signal, and activated by phosphorylated IKK α And IKK *ß* (Kawai and Akira., 2007). TRF signal is a separate branch of TLR signal, which can only be continued through TLR3 and TLR4, where TRIF interacts with TRAF3 and TRAF6 (Akira et al., 2006). In this study, the protein and gene of TLR pathway related factors were determined. It was found that PM_2.5_ can increase the expression of TLR2/4→MyD88→IRAK1 (TRAF6) →TAK1→IKKβ→NF-κB protein and gene in TLR pathway and eventually lead to inflammation (Figure 9). When PHBA, PHPAA and the mixture of PHBA and PHPAA (1:1) were added to PM2.5-stimulated RAW264.7 cells, the above three factors could reduce the expression of TLRs related pathway proteins and genes. These results indicate that PHBA and PHPAA can reduce inflammation caused by PM2.5 by regulating TLRs and their related pathway signal transduction factors.

In this study, we measured the protein and gene of TLR pathway related factors and found that PM_2.5_ can increase the protein and gene expression of TLR pathway and eventually lead to inflammation. PHBA, PHPAA and the mixture of PHBA and PHPAA (1:1) can reduce the expression of TLRs related pathway proteins and genes of RAW264.7 cells stimulated by PM_2.5_, indicating that PHBA and PHPAA can reduce the inflammation produced by PM_2.5_ by regulating TLRs and its related pathway signal transduction factors.

## 5 Conclusion

PM_2.5_ can produce inflammatory reaction and secrete inflammatory cytokines. The molecular mechanism of inflammation produced by PM_2.5_ is related to TLRs and its related pathways, and TLRs pathway can become a potential new target for treating inflammation produced by PM2.5. PHBA, PHPAA and their combination can reduce the inflammatory reaction produced by PM_2.5_. The mechanism of action is related to the inhibition of TLRs and its related pathways, indicating that TLRs signal pathway may be a potential pathway for PHBA and PHPAA to treat inflammation induced by PM_2.5_.

## Data Availability

The original contributions presented in the study are included in the article/Supplementary Material, further inquiries can be directed to the corresponding author.
